# Supplementary material for: Plasma concentration of neurofilament light chain protein decreases after switching from tenofovir disoproxil fumarate to tenofovir alafenamide fumarate
Source: PLoS One. 2019 Dec 11;14(12):e0226276. doi: 10.1371/journal.pone.0226276 (PMC6905536; doi:10.1371/journal.pone.0226276)
Supplement: Attachment — Submitted filename: Rebuttal letter PLoS One 191001.docx [file pone.0226276.s002.docx]

Response to reviewers,

Thanks. The paper has been revised in light of the editorial and reviewers' comments, see below.

There are no restrictions to share a de-identified data set and a data file has been uploaded as Supporting Information file.

*Reviewer #1: Authors report on a surrogate maker, a neurofilament protein, in the plasma to report on neurotoxicity induced by HIV replication. They compare neurotoxicity under tenofovir treatment with toxicity under TAF treatment. Analysis is well done but conclusions should be downplayed due to the lack of a negative control group, different sizes in the groups affecting the significance level, lack of measurement in CSF or orthogonal measurement of HIV replication versus drug induced neurotoxicity. The conclusion that switching to TAF does not increase neurotoxicity compared to tenofovir treatment, using this indirect method, can be made but the discussion and conclusions on the so-called significant decrease in the filament level is not substantiated. It seems that plasma levels were normal and remain normal. Conclusions should be rewritten and re-discussed. Do authors plan to use other methods to investigate HIV replication in the brain in more detail?*

**We agree that results have to be interpreted with large caution, the clinical significance of the highly statistical significant decrease in plasma NFL in the TAF-arm is unclear. This uncertainty is expressed in the conclusions of the abstract: *“It is unclear whether the small decrease in plasma NfL found after switch to TAF is of any clinical relevance, particularly with plasma NfL levels in both arms remaining within the limits found in HIV-negative controls.”* and in the conclusions: “*The clinical significance of, and the mechanisms behind, this decrease within the normal range, are unclear*”.**

**It is not possible to investigate CNS HIV replication or other CNS measurements in the present study. Included as a weakness in discussion, row 245-247. However, other studies with CSF sampling are ongoing.**

*Reviewer #2: This a post-hoc analysis of the Gilead GS-US-292-0109 trial restricted to people who were on E/C/F/TDF. This has been done so that the only difference between the arms would be using TAF instead of TDF after switching with the rest of the drugs in the regimen being identical. Thus, a cleaner dataset in which any difference in markers after switching could be attributed to TAF vs. TDF. Because, people originally enrolled in the trial were at time zero randomized either to stay on TDF or switch to TAF, exchangeability is also retained in this subset of data which was included for analysis. The aim was to investigate whether switching to TAF was associated with increased neuronal injury compared to remaining on TDF. The manuscript is clearly written but there are few main points that need to be addressed:*

*1. Although groups were exchangeable at baseline, out of the 459 who were on E/C/F/TDF at baseline, only 414 had week 84 samples/values so there is potential attrition bias in this analysis. It is crucial to address this issue in more details than what was done by the authors in the present form:*

*i) Table 1 should show all potential common causes of treatment allocation and risk of neuronal injury. Ideally such a table should be identical to that shown in the parental paper published in Lancet Infectious Diseases [Lancet Infect Dis. 2016 Jan;16(1):43-52]. This is to show that attrition did not create imbalances in important potential confounders.*

**Good point! Table 1 has been changed accordingly, no imbalances were found except for ethnic origin (Hispanic or Latino origin) with a similar difference between groups that were found in the full main study – added to Results row 139-141. We also compared demographics in the 43 patients who did not meet the inclusion criteria with the 416 included and found no differences, row 142-144.**

*ii) If imbalances in important confounders are detected, multivariable analysis should be conducted to control for these. Ideally, this analysis should be performed using marginal methods evaluating the effect of treatment after averaging out the effect of the covariates. This is because authors are interested in the causal effect of switching to TAF if there was no attrition bias. Specifically, marginal weighted linear regressions models should be employed.*

**No imbalances of importance found**

*iii) Attrition bias should be clearly reported in the Figures by adding a footonote with the number of people contributing measurements at the 24 and 84 week time points.*

**Number of people contributing measurements at each time point added to legends of figure1 and 2.**

*iv) The issue of potential informative censoring and consequent attrition bias (as people who are retained in TAF up to week 84 are likely to be those who have less injury) needs also to be discussed as a limitation.*

**This has been added as a limitation, rows 248-250**

*v) Authors should explain why numbers in Table and in the text are inconsistent (416 total vs 414 in the text and abstract, 144 in the TDF arm vs. 142 in the text)*

**Thanks for noticing, the correct numbers should be 416 and 144, has been changed.**

*vi) Authors should also discuss power and potential issues related to the chosen target population in the trial. Indeed, contrary to what shown in the parental paper, there was no improvement in renal function in the TAF arm in this subset. This shades doubt also on the comparison of the risk of neuronal injury.*

**This must be a misunderstanding, eGFR and creatinine improved in the TAF-arm and was significantly different compared to the TDF-arm after 84 weeks, see row 175-182 and figure 2, we have thus no doubt that the significant difference between the arms is real. We recognize that the 5.8 % increase of plasma NfL in the numerically smaller TDF-arm is close to significant and thus possibly underpowered, but on the other hand, based on data from HIV-negative controls (ref 18) plasma NFL could be expected to increase 5.3 % by 84 weeks of ageing. We have included a short discussion about this in the Abstract, rows 39-40 and in Results, rows 160-162.**

*vii) Clinical relevance of the small difference found is rightly questioned in the abstract. A discussion of this point should be included also in the main text.*

**Discussion of clinical significance added, row 237-239. Also mentioned in conclusions rows 254-255**

*Reviewer #3: The hipothesis of your study is interesting and your conclusion supported the idea that TAF was a drugs with lower toxicity than TDF.*

*Some little revision are however due before publish your paper*

*In tab 1 you present data registered at baseline. Could be better to show in tab any difference within the two group also regarding age creatinine and nfl showing the p value.*

**P-values added to the table.**

*It is possible that the different number of patients on TAF anf TDF (2:1) could modify the statistical significance of your results: really the difference between Nfl or creatinine could be modified by study designe and patients distribution into the the two arms. Please discuss about any possible bias linked to this aspect.*

**We agree that there is less power to detect changes in the smaller TDF group, but the slight plasma NfL increase in the TDF group is properly compared to the slight decrease in TAF group and found convincingly significant. The other part of the comment is addressed in answers to Reviewer #2**

*At the same time the value of creatinine could be not perfectly associated to the real filtration capacity of every patient. A GFR calculation could give us a more exact estimation of renal function.*

**Agree. We have included eGFR results, see rows 175-182, and 195-196. The rationale for using creatinine rather than eGFR when associations with NFL are analysed is that NFL is strongly correlated with age and age is one of the factors used when eGFR is calculated.**

*Finally the figures showed a different trend of Nfl concentrations at 24 and 84 weeks, mainly in TDF group. Could you speculate about this different bifasic dynamic?*

**This is very hard to speculate about, the only explanation we have is that changes, whatever their mechanisms are, take longer than 24 weeks to develop.**

*Reviewer #4: This is a well-thought through and well-written paper, on an interesting and clinically relevant hypothesis.*

*1. Line 35: for 10.3 to 9.6 pg/mL: are these the median values? or mean? Need to state. The values presented here are different from the values presented in the main text Results section(9.3 to 8.8 pg/mL): was one set of values: median, and one set of values: mean?*

**Thanks for noticing, values in abstract are wrong and we have changed them. Values are presented as geometric means which has been clarified in the abstract.**

*Line 37: Once again the reported change in plasma NfL in the abstract (11.1 to 11.7pg/mL) is different from what is reported in the main test results (9.7 to 10.2pg/mL). When you say 'at follow up', do you mean week 84?*

**See above. Follow-up changed to week 84.**

*2. Line 36: Does the p<0.01 value relate to the comparison of the percentage change in both arms? If so, you should state that; this sentence is very confusing to read. Also worth stating the median/mean percentage change in both arms at week 84, to put that p-value into context.*

**We have included percentage changes in both arms with CI95%. The p-value refers to the difference between groups which has been clarified.**

*3. Line 114: How many times were each sample analysed? Were the samples analysed in duplicate/triplicate?*

**Samples were analysed once, added to row 119**

*4. Line 135: Table 1 does not show the p-values for statistically significant differences in age and gender, so the reference to Table 1 should be shifted to the sentence before. Referencing Table 1 here implies you are demonstrating p-values for differences in age and gender in Table 1.*

**Agree, we have changed the text before table 1 is referenced and P-values have been included in table 1.**

*Line 142: What is the p-value for difference in plasma NfL between the 2 arms at baseline? Since you describe the p-values, you should put it in Table 1.*

**There was no difference in plasma NfL at baseline between the groups, see row 155-156**

*5: Line 148: What other factors did you look at for associations with plasma NfL? Other factors related to eGFR perhaps: gender, ethnicity, weight?*

**No correlation between plasma NfL and gender or ethnicity, added at row 164.**

*6: Line 219: to also add the lack of age-related reference values for plasma NfL- unlike CSF NFL which has age-related reference values, and thus may be a more reliable/ interpretable biomarker.*

*7: The lack of concurrent CSF sample needs to be acknowledged in the discussion, given that plasma NfL using Simoa is still novel, and interpretation is more difficult for the reasons mentioned above*

**Included as weaknesses in discussion, rows 245-247.**

*8: I agree with the final conclusions drawn from this paper.*

**Thanks!**

Sincerely,

Magnus Gisslén

Dpt of Infectious Diseases

The Sahlgrenska Academy

University of Gothenburg

SE-416 85 Gothenburg

Sweden

Tel: +46 31 343 55 22

E-mail: [magnus.gisslen@gu.se](mailto:magnus.gisslen@gu.se)
